# Design of a Capacitive MEMS Accelerometer with Softened Beams

**DOI:** 10.3390/mi13030459

**Published:** 2022-03-17

**Authors:** Chenggang Wang, Yongcun Hao, Zheng Sun, Luhan Zu, Weizheng Yuan, Honglong Chang

**Affiliations:** 1 Ningbo Institute of Northwestern Polytechnical University, Ningbo 315103, China; wangchenggang0510@mail.nwpu.edu.cn (C.W.); changhl@nwpu.edu.cn (H.C.); 2 MOE Key Laboratory of Micro and Nano Systems for Aerospace, Northwestern Polytechnical University, Xi’an 710072, China; sun.zheng@mail.nwpu.edu.cn (Z.S.); zuluhan@mail.nwpu.edu.cn (L.Z.); yuanwz@nwpu.edu.cn (W.Y.)

**Keywords:** MEMS, accelerometer, electrostatic assembly, stiffness softening

## Abstract

Lower stiffness can improve the performance of capacitive-based microelectromechanical systems sensors. In this paper, softened beams, achieved by the electrostatic assembly approach, are proposed to lower the stiffness of a capacitive MEMS accelerometer. The experiments show that the stiffness of the accelerometer is reduced by 43% with softened beams and the sensitivity is increased by 72.6%. As a result, the noise of the accelerometer is reduced to 26.2 μg/√Hz with an improvement of 44.5%, and bias instability is reduced to 5.05 μg with an enhancement of 38.7%. The electrostatic assembly-based stiffness softening technique is proven to be effective and can be used in many types of MEMS devices.

## 1. Introduction

The capacitive-based microelectromechanical systems (MEMS) accelerometer, as a typical inertial sensor, has been widely used in consumer electronics, industrial applications, and inertial navigation [1,2,3]. However, performance shortages remain a bottleneck for further applications. Sensitivity is the key factor that influences the performance of accelerometers. Higher sensitivity delivers better performance, and the sensitivity of the accelerometer is inversely proportional to the square of its frequency [4]. There are two basic ways to enhance the sensitivity: increasing the proof mass and reducing the stiffness. The proof mass can be increased by enlarging volume [5] and density [6,7]. The stiffness can be reduced by thinning the width of the elastic beams [8] and by multiplying the folding beams [9,10]. However, due to the limitations of MEMS manufacturing technology, these approaches are no longer effective.

Recently, geometric nonlinear theory was applied to capacitive-based MEMS sensors to enhance their performance. In 2016, the University of Glasgow first reported a MEMS gravimeter with curved beams in *Nature* [11]. In the paper, the curved beams were softened by gravity and their stiffness was reduced dramatically. As a result, the gravimeter increased in sensitivity and could measure Earth’s tides successfully. Next, more MEMS sensors with softened curved beams were created [12,13,14]. The principle of geometric nonlinearity is that an elastic beam would soften under an axial compressive load, resulting in a reduction in stiffness. The axial compressive load is the precondition for softening the elastic beam. In most cases, the compressive load is provided using the gravity assembly and mechanical assembly approaches. Such approaches are effective but not sufficiently flexible because the assembly loads are constant. Specifically, the assembly approaches fail to adjust the axial load to compensate for fabrication errors. Therefore, it is necessary to find a more flexible approach to assemble and soften the elastic beam.

In this paper, the electrostatic assembly approach is adopted to soften the elastic beam of a capacitive MEMS accelerometer. Unlike the gravity assembly and mechanical assembly, the electrostatic assembly can regulate the axial load dynamically through the assembly voltage. Using this method, the elastic beam is softened successfully and the stiffness of the accelerometer is reduced to 11.6 N/m from 20.2 N/m, and the sensitivity of the accelerometer is enhanced by 72.6%. As a result, the noise and bias instability is promoted to 26.2 μg/√Hz and 5.05 μg, respectively. The proposed accelerometer is fabricated using a silicon-on-insulator (SOI)-based dicing-free process and tested by a capacitive-voltage converter (CVC) readout circuit.

## 2. Design

### 2.1. Stiffness Softening

**Figure 1:**
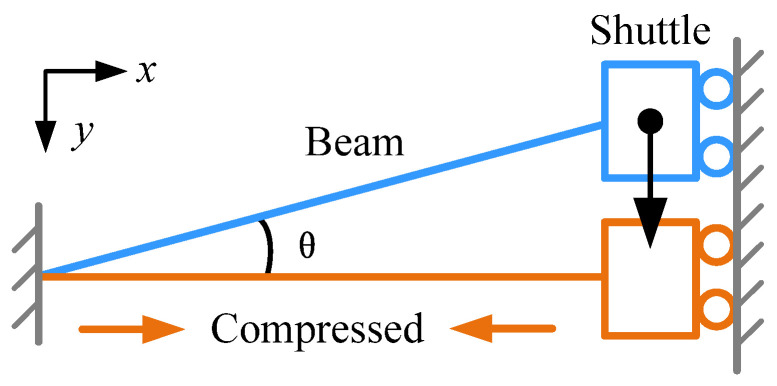
Schematic diagram of stiffness softening.

According to geometric nonlinear theory, an elastic beam would soften under an axial compressive load, called “stiffness softening”. As a result, such an elastic beam is also called a “nonlinear beam”. Referring to Figure 1, suppose there is a nonlinear beam with one end fixed and the other end guided by a shuttle. The initial angle of the beam from the *x*-axis is *θ*. When the shuttle moves along the *y*-axis under an external load, the beam is compressed. In the horizontal position, the compression energy of the beam reaches its maximum capacity and the axial compressive load can be expressed as:

(1) $$F_{c}=EA\left(1-\cos\theta \right),$$

where *E* is Young’s modulus, *A* is the cross-sectional area of the beam, and *θ* is the angle of the nonlinear beam from the *x*-axis.

When the shuttle moves away slightly from the horizontal position, the released compression energy of the beam compensates for the bending energy and lowers the bending stiffness. The bending stiffness of the compressed beam can be expressed as [15]:

(2) $$k_{n}=\frac{Eh_{n}b_{n}^{3}}{l_{n}^{3}}-\frac{12F_{c}}{\pi^{2}l_{n}}=\frac{Eh_{n}b_{n}^{3}}{l_{n}^{3}}-\frac{12EA}{\pi^{2}l_{n}}\left(1-\cos\theta \right),$$

where *h_n_*, *b_n_*, and *l_n_* are the thickness, width, and length of the beam, respectively. The axial compressive load is the precondition of stiffness softening and is achieved by the electrostatic assembly approach in this paper.

### 2.2. Accelerometer Design

**Figure 2:**
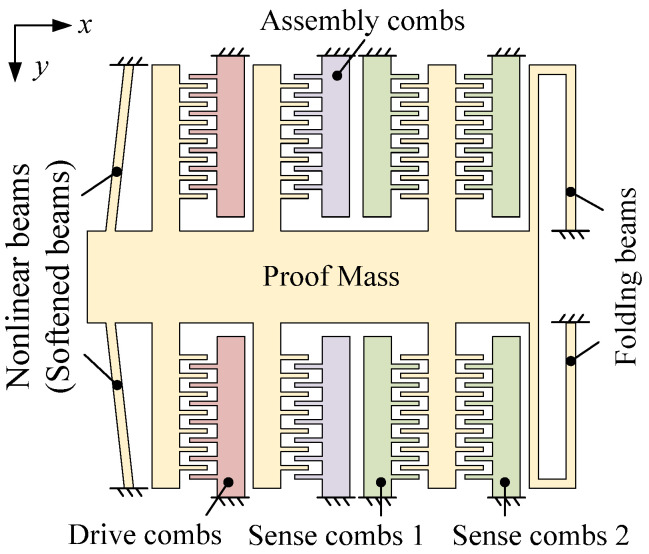
A schematic design of the sensor.

The proposed capacitive accelerometer, as shown in Figure 2, comprises the proof mass, nonlinear beams (labeled “softened beams” after assembly), folding beams, assembly combs, drive combs, and sense combs. The nonlinear beams and the folding beams are used to support the proof mass. The assembly combs are designed to assemble the accelerometer. When DC voltage is applied to the assembly combs, the proof mass moves along the *x*-axis to soften the nonlinear beams. Thus, the nonlinear beams are softened. The drive combs are used to excite the proof mass in the response experiment, and the sense combs are used to detect the displacement of the proof mass. There is no difference in structure between the drive combs and the assembly combs except for the quantity. There are two types of sense combs: sense combs 1 and sense combs 2. When the accelerometer moves in the *x*-direction, the capacitance of sense combs 1 decreases but the capacitance of sense combs 1 increases. Thus, sense combs 1 and sense combs 2 make a differential configuration. The common-mode noise has the same influence on sense combs 1 and sense combs 2, and it can be eliminated by the differential operation.

The mechanical sensitivity of the accelerometer is defined as the displacement of the proof mass under acceleration, which can be expressed as [13]:

(3) $$S=\frac{m}{k}=\frac{1}{\omega^{2}},$$

where *m*, *k*, and *ω* are the proof mass, stiffness, and the resonant frequency of the accelerometer, respectively. Hence, a reduction of the stiffness can effectively enhance the sensitivity of the accelerometer.

The total stiffness of the accelerometer is determined by the nonlinear beams and the folding beams. The folding beam is a common structure used in MEMS devices and its bending stiffness *k_f_* equals

(4) $$k_{f}=\frac{Eh_{f}b_{f}^{3}}{2l_{f}^{3}},$$

where *h_f_*, *b_f_*, and *l_f_* are the thickness, width, and length of the folding beam, respectively. Thus, the total stiffness of the proposed accelerometer equals

(5) $$k=2(k_{n}+k_{f})=\frac{2Eh_{n}b_{n}^{3}}{l_{n}^{3}}+\frac{Eh_{f}b_{f}^{3}}{l_{f}^{3}}-\frac{24EA}{\pi^{2}l_{n}}\left(1-\cos\theta \right).$$

The resonant frequency of the accelerometer in working mode can be expressed as

(6) $$f=\frac{1}{2\pi }\sqrt{k/m}.$$

**Figure 3:**
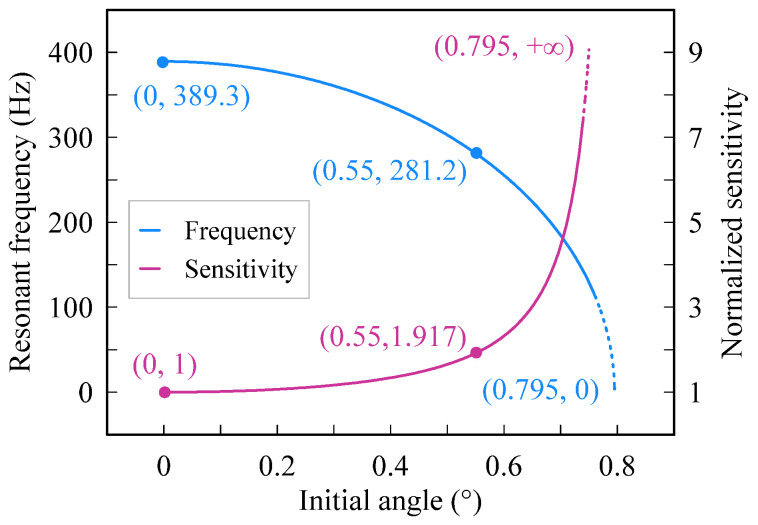
Variation of resonant frequency and sensitivity as initial angle changes.

As Equations (5) and (6) imply, the resonant frequency of the accelerometer in working mode is related to the initial angle of the nonlinear beam. Using the parameters listed in Table 1, the relationship between the initial angle and the resonant frequency is demonstrated in Figure 3. The resonant frequency tends to decrease as the initial position increases. The resonant frequency is 389.3 Hz when the initial angle is zero, whereas the resonant frequency is close to zero when the initial angle approximates 0.795 degrees. A bigger initial angle helps reduce the resonant frequency; however, it needs a considerable assembly force. In our design, an initial angle of 0.55 degrees is chosen. As a consequence, the resonant frequency of the accelerometer would drop from 389.3 Hz to 281.2 Hz when the shuttle moves from the initial position to the horizontal position. The normalized sensitivity of the accelerometer tends to enhance as the initial angle increases. As shown in Figure 3, the sensitivity of the accelerometer is 1 when the initial angle is zero, whereas the sensitivity tends to infinity when the initial angle approximates 0.795 degrees.

It should be noted that the mechanical sensitivity of the accelerometer varies due to the dispersion of materials properties and the geometrical dimensions. In general, Young’s modulus and beam width are the crucial factors that influence the sensitivity. The most commonly used Young’s modulus for silicon is 169 GPa. However, in practice, it may vary from 165 GPa to 172 GPa. The designed width of the folding beam and the nonlinear beam is 25 μm. However, the beam width likely varies from 24 μm to 26 μm due to fabrication errors. If the above factors are taken into account, then based on Equations (3) and (5), the sensitivity of the accelerometer would vary from 26.7 nm/g to 41.1 nm/g.

## 3. Fabrication

**Figure 4:**
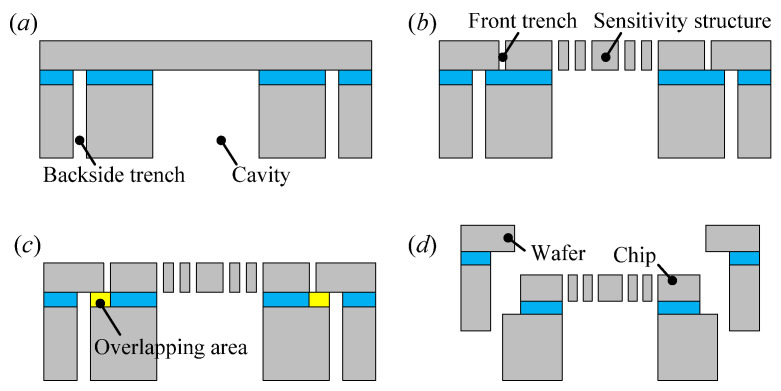
SOI-based dicing-free process. (**a**) Patterning of substrate layer. (**b**) Patterning of structure layer. (**c**) Etching of overlapping area. (**d**) Chip unloading.

**Figure 5:**
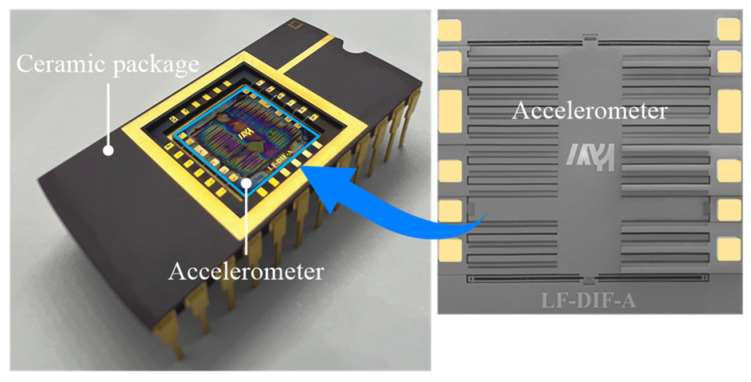
Accelerometer encapsulated by a ceramic package.

The proposed accelerometer was fabricated using an SOI-based dicing-free process [16]. As shown in Figure 4, the fabrication process comprises four steps. First, the substrate layer of the SOI wafer is patterned with a cavity and a backside trench by the lithography and deep reactive ion etching process; then, the bared oxide is removed from the backside using the buffered oxide etch (BOE) solution. Second, the structure layer is patterned with the sensitivity structure and the front trench. The overlapping area that connects the accelerometer chip and the SOI wafer is formed between the front trench and the backside trench. Third, the oxide of the overlapping area is removed using the BOE resolution from both sides. Finally, the accelerometer is unloaded from the SOI wafer to complete the fabrication. As shown in Figure 5, the fabricated accelerometer is encapsulated by a ceramic package to facilitate the subsequent measurements.

## 4. Experiment Schemes

### 4.1. Frequency Response

**Figure 6:**
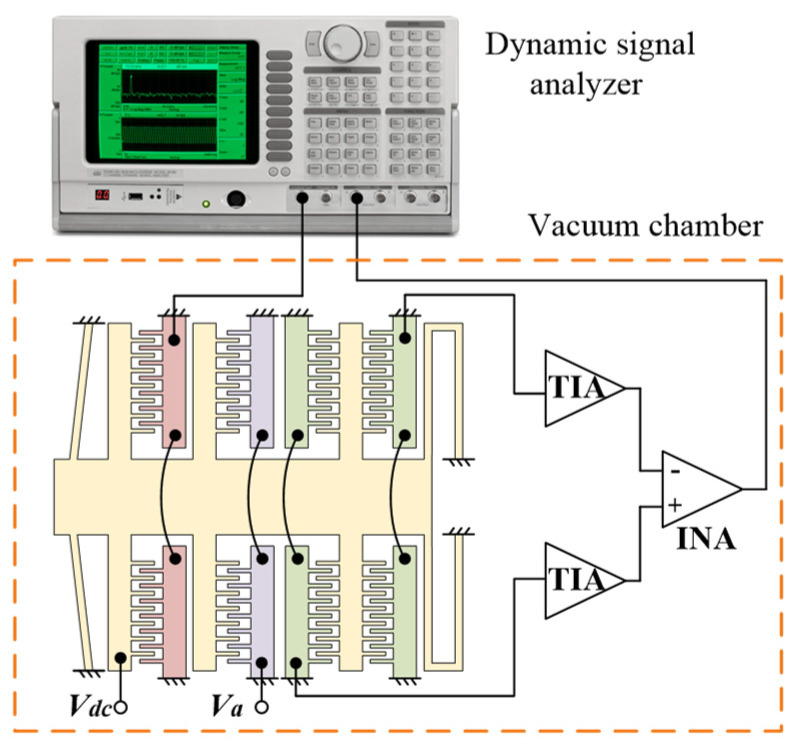
Frequency response circuit.

**Figure 7:**
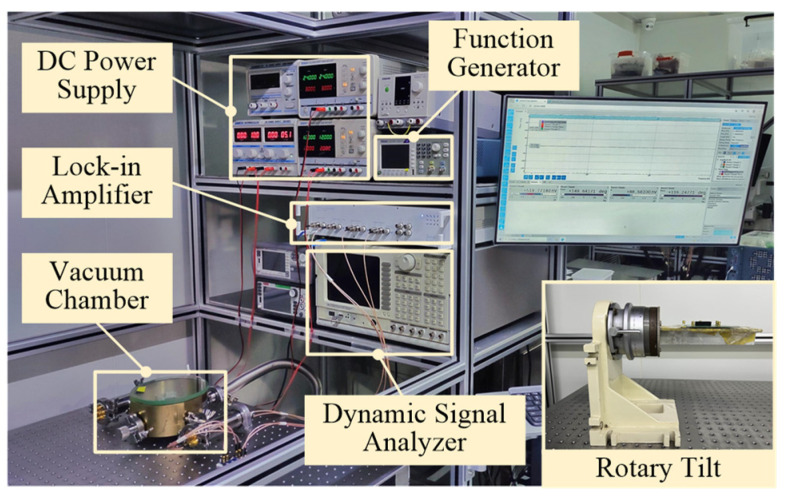
Experiment platform and apparatus.

The resonant frequency of the fabricated accelerometer was measured in a vacuum chamber using the frequency response circuit. As shown in Figure 6, the driving signal, provided by the dynamic signal analyzer Stanford Research System Model SR785 (Stanford Research Systems, Sunnyvale, CA, USA) is applied on the drive combs to actuate the proof mass. The displacement of the proof mass is picked up by the differential sense combs. The electric currents generated in the sense combs are converted to voltage signals by the transimpedance amplifier. Finally, the signal returns to the dynamic signal analyzer after being differenced by an instrumentation amplifier. Two DC power supplies MYWAVE MPD-3303S (Shenzhen MYWAVE Instrument Co., Shenzhen, China) and ZHAOXIN KXN-3001D (Shenzhen Zhaoxin Electronic Instruments & Equipment Co., Shenzhen, China) were used to provide the bias voltage (*V_dc_* = 5 V) and the assembly voltage (*V_a_*), respectively. The experiment platform and apparatus are shown in Figure 7.

### 4.2. Performance Testing

**Figure 8:**
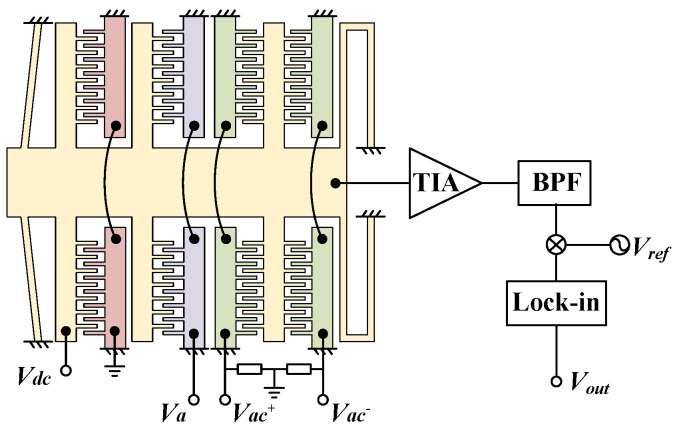
Schematic of the CVC readout circuit.

The proposed accelerometer was encapsulated at atmospheric pressure in a ceramic package and mounted on a rotary tilt to evaluate its performance via a capacitance-to-voltage converter (CVC) readout circuit. As shown in Figure 8, two high-frequency carriers, *V_ac_^+^* and *V_ac_^-^*, with the same amplitude but opposite phase, are applied on the differential sensor combs. The proof mass produces a differential capacitance of *C*_0_ with the differential sense combs. Under an external acceleration, the proof mass moves and causes a capacitance variation of Δ*C*. The variation in capacitances is modulated by the carrier to output a current. Then, the current is converted to a voltage signal via a transimpedance amplifier and filtered by a bandpass filter. Finally, the signal is demodulated and low-pass filtered by a lock-in amplifier. The output voltage *V_out_* of the readout circuit equals

(7) $$V_{out}=\frac{\Delta C}{C_{0}}G,$$

where is G is the total gain of the CVC readout circuit.

As shown in Figure 7, a function generator (Tektronix AFG1062, Tektronix Inc., Beaverton, OR, USA) is used to provide the high-frequency carrier. A lock-in amplifier (Zurich Instruments HF2LI, Zurich Instruments, Zurich, Switzerland) is adopted to implement the phase multiplication demodulation and low-pass filtering. Additionally, the DC power supply ZHAOXIN KXN-3001D (Shenzhen Zhaoxin Electronic Instruments & Equipment Co., Shenzhen, China) is adopted to supply the assembly voltage.

## 5. Experiment Results

### 5.1. Resonant Frequency

**Figure 9:**
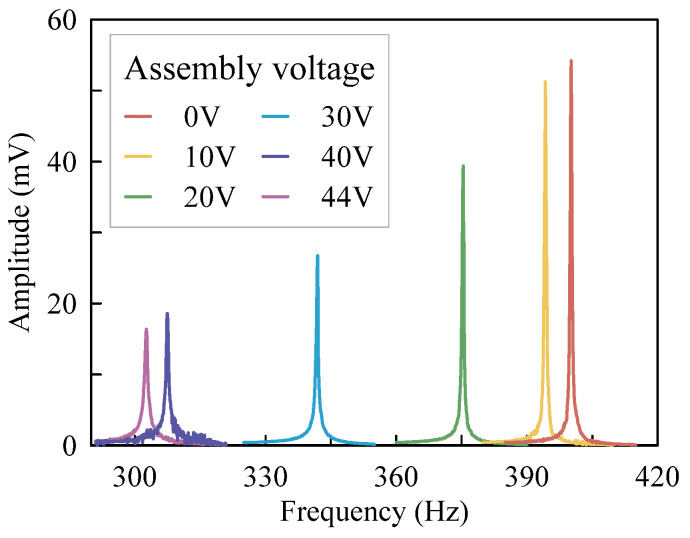
Frequency responses of the accelerometer.

**Figure 10:**
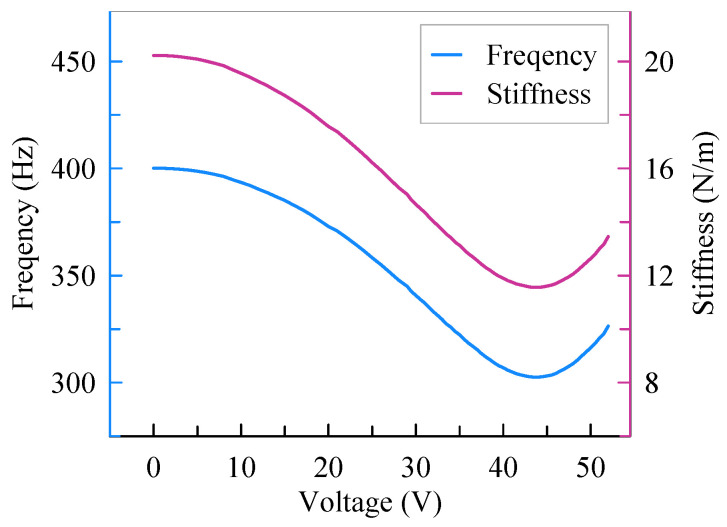
Frequency and stiffness variation.

The frequency responses of the accelerometer with different assembly voltages are shown in Figure 9. The initial resonant frequency is 400.1 Hz before assembly. As the assembly voltage increases to 44 V, the resonant frequency drops to a minimum of 302.5 Hz. By this time, the shuttle is in the horizontal position. When the assembly voltage continues to increase, the shuttle keeps moving and the resonant frequency increases for the releasing of the axial load. Thus, as shown in Figure 10, the resonant frequency begins to rise as the assembly voltage exceeds 44 V. The resonant frequency variation is caused by the stiffness of the accelerometer because the proof mass is constant. Thus, the variation trend of stiffness is coincident with the resonant frequency. As illustrated in Figure 10, the stiffness of the accelerometer is 20.2 N/m before assembly and then decreases to 11.6 N/m with an assembly voltage of 44 V. That is, the stiffness of the accelerometer is reduced by 42.6% with the softened beams. In this experiment, the drive combs aim to drive the proof mass to vibrate while the assembly combs provide a static displacement for the proof mass. In theory, the drive combs can contribute to an assembly force. However, the assembly force caused by the drive combs is much smaller than that of assembly combs because the voltage applied on the drive combs is an alternating voltage of 100 mV. Thus, the assembly force caused by the drive combs can be neglected.

### 5.2. Sensitivity, Range, and Bandwidth

The accelerometer is mounted on the rotary tilt to measure its sensitivity. In the experiment, the rotary tilt is rotated to 15 degrees from the horizontal position with a step of 1 degree. The effective gravity acting on the proof mass of the accelerometer is

(8) a=g*sinβ,

where g is the gravitational acceleration and β is the rotary angle. Thus, the acceleration range is 0 to 0.26 g when the tilt rotates.

**Figure 11:**
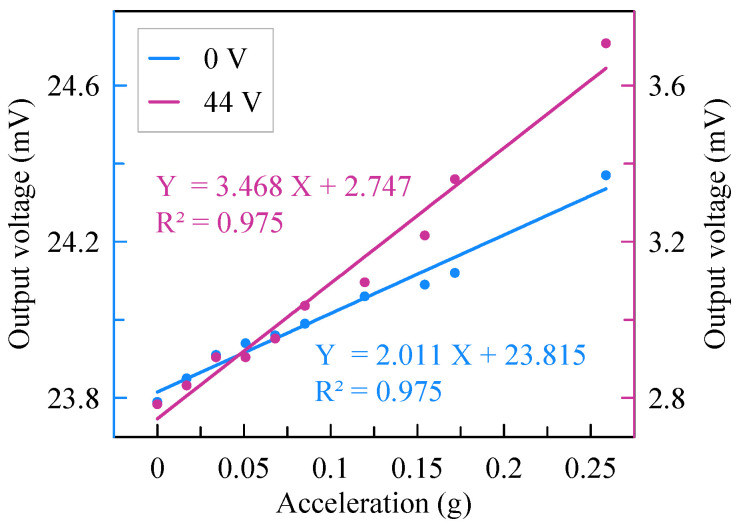
Sensitivity variation of the accelerometer.

The outputs of the readout circuit are recorded by a dynamic signal analyzer when the tilt rotates. As shown in Figure 11, the sensitivity of the accelerometer is 2.01 mV/g before assembly but changes to 3.47 mV/g with an assembly voltage of 44 V. Thus, the sensitivity of the accelerometer is enhanced by 72.6% with the softened beams. The measuring range of the accelerometer is 0.26 g with nonlinearity of 2.5%.

**Figure 12:**
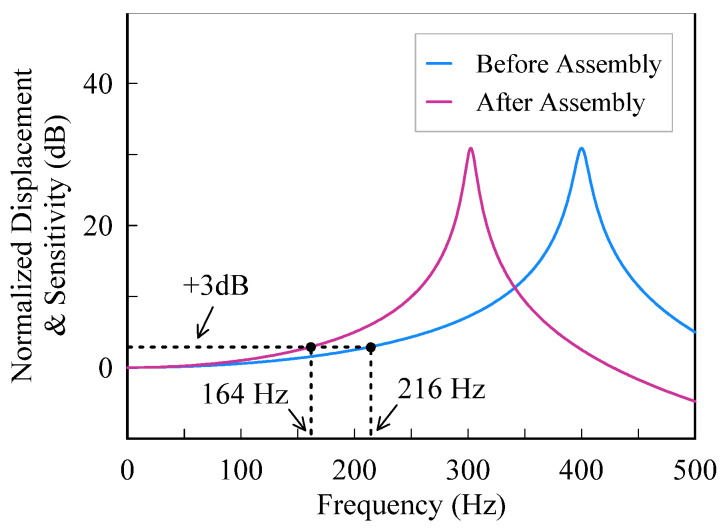
Frequency response of the accelerometer.

The frequency response is calculated based on the kinetic equation of the spring–mass–damper system. The amplitude of the spring–mass–damper system equals

(9) $$X=\frac{x_{0}}{\sqrt{{\left(1-{\left(\frac{f}{f_{n}}\right)}^{2}\right)}^{2}+{\left(2\xi \frac{f}{f_{n}}\right)}^{2}}},$$

where *f* is the input frequency, *f_n_* is the resonant frequency, *ξ* is the damping ratio, and *x*_0_ is the static displacement. Using the measured data and based on Equation (9), the frequency response curve is plotted in Figure 12. The cut-off frequency before and after assembly is 216 Hz and 164 Hz, respectively. In other words, the bandwidth of the accelerometer drops from 216 Hz to 164 Hz after assembly.

### 5.3. Noise and Bias Instability

**Figure 13:**
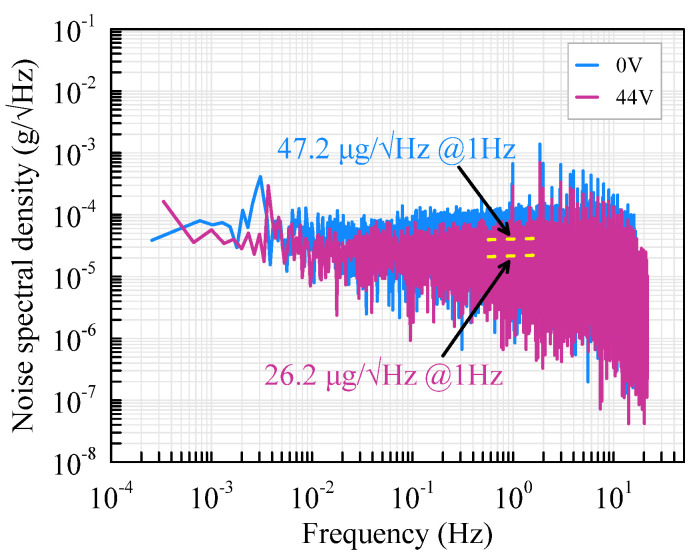
Noise variation of the accelerometer.

The output voltage of the accelerometer is recorded by the lock-in amplifier for more than 40 min with a sampling frequency of 225 Hz. The voltage noise is acquired by calculating the power spectral density of the output voltage. The noise of the accelerometer equals voltage noise divided by the sensitivity. As demonstrated in Figure 13, the noise of the accelerometer is 47.2 μg/√Hz at 1 Hz before assembly but is promoted to 26.2 μg/√Hz with an assembly voltage of 44 V. Therefore, the noise of the accelerometer is reduced by 44.5% with softened beams.

**Figure 14:**
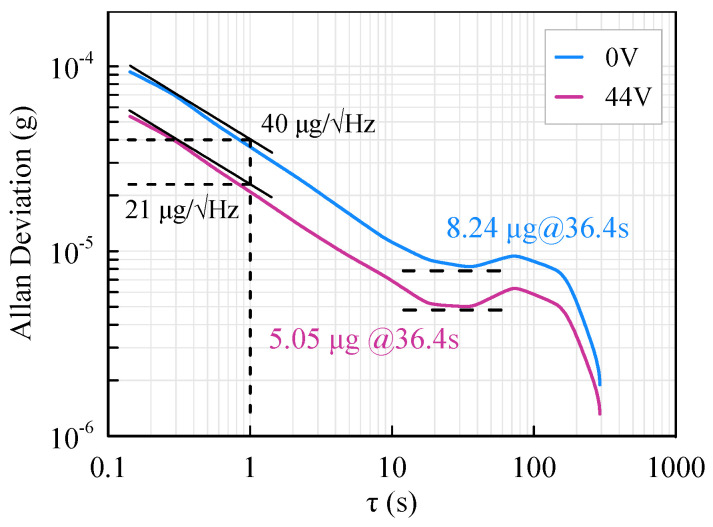
Allan deviation of the accelerometer.

The bias instability of the accelerometer is characterized by the Allan deviation. As shown in Figure 14, the bias instability of the accelerometer is 8.24 μg before assembly but is promoted to 5.05 μg with an assembly voltage of 44 V. Thus, the bias instability of the accelerometer is reduced by 38.7% with the softened beams. The noise of the accelerometer can also be characterized via the Allan deviation. Different from the noise spectral density method, the Allan deviation gives a more theoretical characterization of the noise floor. Using the Allan deviation method, the noise of the accelerometer is 40 µg/√Hz before assembly and 21 μg/√Hz after assembly. The noise represented by Allan deviation matches well with that of the power spectral density approach.

## 6. Conclusions

This paper proposed a capacitive MEMS accelerometer to verify the feasibility of softened beams in performance enhancement. The electrostatic assembly method was used to soften the elastic beams. Using this method, the stiffness of the accelerometer was reduced by 42.6%, leading to a sensitivity increase of 72.6%. As a result, the noise and bias instability were improved by 44.5% and 38.7%, respectively. The electrostatic assembly approach is more flexible and more precise than the gravity assembly and mechanical assembly approaches because the assembly voltage can easily be regulated. However, the electrostatic assembly approach may degrade the noise and bias instability in the fluctuation of the assembly voltage. Therefore, an extra voltage regulator circuit is needed to stabilize the assembly voltage in future work. In addition, the stiffness of a nonlinear beam can be reduced by decreasing the width, increasing the length, and enlarging the initial angle. That is, the overall performance of the accelerometer can be further enhanced.

## Figures and Tables

**Table 1:** Parameters of the proposed accelerometer.

Parameters	Value
Young’s modulus of silicon (E)	169 GPa
Proof mass (m)	3.2 μg
Thickness of folding beam ($h_{f}$)	60 μm
Width of folding beam (*b_f_*)	25 μm
Length of folding beam (*l_f_*)	2650 μm
Initial angle of nonlinear beam (θ)	0.55°
Cross-sectional area of nonlinear beam (A)	1500 μm^2^
Thickness of nonlinear beam ($h_{n}$)	60 μm
Width of nonlinear beam (*b_n_*)	25 μm
Length of nonlinear beam (*l_n_*)	3100 μm
Number of drive combs	240
Number of assembly combs	960
Gap of combs	4 μm
Sense capacitance	26.8 pF
Overall dimension	8.8 mm × 8.8 mm
